# Shivering during targeted temperature management after cardiac arrest: a physiologic description

**DOI:** 10.1186/cc13688

**Published:** 2014-03-17

**Authors:** T May, DS Seder, GF Fraser, CF Hoover, RR Riker, BM McCrum

**Affiliations:** 1 Maine Medical Center, Portland, ME, USA

## Introduction

Targeted temperature management (TTM) is used to treat hypoxic ischemic encephalopathy (HIE), elevated intracranial pressure, status epilepticus, and other brain injuries. Shivering complicates TTM, and the associated energy burden and its response to treatment are poorly understood. We describe the pattern of shivering and response to neuromuscular blockade in a series of patients undergoing TTM after cardiac arrest using continuous indirect calorimetry.

## Methods

With IRB approval, we studied 16 patients undergoing TTM for HIE with continuous calorimetry during their treatment. The calorimeter measures inspired and expired oxygen and carbon dioxide, which are used in the Weir equation to calculate resting energy expenditure (REE). We excluded patients known to be actively seizing, requiring FiO_2 _>0.5, with early spasticity, or who did not shiver. All patients received counterwarming, moderate analgosedation, and bolused vecuronium in response to visible shivering, measured hourly by nurses using the Bedside Shivering Assessment Scale.

## Results

Sixteen patients of average age 57 included 12 men. Twelve patients had CPC of 1 to 3 on hospital discharge. Sixteen shivering events were monitored with calorimetry among the 12 patients. The average rate of energy expenditure (without shivering) leading up to and following paralytic treatment was 1,425 kcal/hour (± 489 kcal/ hour) and 1,386 kcal/hour (± 235 kcal/hour). This was significantly greater than the predicted basal energy expenditure (1,089 ± 222 kcal; *P *= 0.007 and *P *< 0.001). Time from a change in baseline energy expenditure to recognition and treatment of clinical shivering was 57 (± 64) minutes, and from treatment with neuromuscular blockade to baseline energy expenditure was 30 (± 20) minutes. This accounts for a total difference of 15,223 kcal (± 10,997 kcal) before treatment and 7,113 kcal (± 3,706 kcal) after treatment for each shivering episode compared with baseline (*P *= 0.01 and *P *= 0.003).

## Conclusion

The energy burden of shivering is underestimated by standard nutritional formulas in patients undergoing TTM after cardiac arrest. Subclinical shivering is associated with increased energy expenditure. Clinical recognition occurs long after the increase in metabolic activity, and persists for a significant period of time after treatment. These findings should influence how shivering is monitored and treated during TTM.

